# Environmental Impact of Meals: How Big Is the Carbon Footprint in the School Canteens?

**DOI:** 10.3390/foods11020193

**Published:** 2022-01-12

**Authors:** Mirco Volanti, Francesco Arfelli, Esmeralda Neri, Aurora Saliani, Fabrizio Passarini, Ivano Vassura, Gianluca Cristallo

**Affiliations:** 1 Department of Industrial Chemistry “Toso Montanari”, University of Bologna, Viale del Risorgimento 4, 40136 Bologna, Italy; mirco.volanti@unibo.it (M.V.); francesco.arfelli3@unibo.it (F.A.); esmeralda.neri1988@gmail.com (E.N.); aurora.saliani@gmail.com (A.S.); ivano.vassura@unibo.it (I.V.); 2 Interdepartmental Centre of Industrial Research “Renewable Resources, Environment, Sea ad Energy”, University of Bologna, Via Angherà 22, 47922 Rimini, Italy; 3 CAMST Soc. Coop. A R.L.—La Ristorazione Italiana, Via Toscanelli 318, 40121 Bologna, Italy; gialereb@gmail.com

**Keywords:** sustainability, Life Cycle Assessment, carbon footprint, food sector, consumer behaviour

## Abstract

The inhabitants of the world are expected to grow by two billion in the next two decades; as population increases, food demand rises too, leading to more intensive resource exploitation and greater negative externalities related to food production. In this paper the environmental impact of meals provided in school canteens are analysed through the Life Cycle Assessment methodology, in order to evaluate the GHGs emissions released by food production. Meals, and not just individual foods, have been considered so as to include in the analysis the nutritional aspects on which meals are based. Results shows that meat, fish and dairy products are the most impacting in terms of greenhouse gas emissions, with values that shift from 31.7 and 24.1 kg CO_2_ eq for butter and veal, to 2.37 kg CO_2_ eq for the octopus, while vegetables, legumes, fruit and cereals are less carbon intensive (average of 3.71 kg CO_2_ eq for the considered vegetables). When the environmental impact is related to the food energy, the best option are first courses because they combine a low carbon footprint with a high energy content. The results of the work can be used both by the consumer, who can base the meal choice on environmental impact information, and by food services, who can adjust menus to achieve a more sustainable production.

## 1. Introduction

According to the United Nations projections, the world population is expected to grow by 2 billion people by 2050, reaching 9.7 billion individuals worldwide [1]. More people on this planet means higher food demand and, consequently, greater competition for natural resources. Food production relies on the exploitation of natural capital inputs such as water, land, energy and biodiversity, and is responsible for negative externalities as land degradation, water stress and air pollution, contributing to 60% to the terrestrial biodiversity loss [2,3]. Moreover, agriculture both contributes to climate change and is affected by climate change: on one hand it is accounted to release the 24% of greenhouse gases (GHG) in the atmosphere, while on the other hand extreme weather events affect crop yields and food prices [2,3,4]. According to that, current food consumption patterns must be revised in order to improve their sustainability and to reduce the environmental impact of our food system, stimulating the preservation of natural resources, reducing the food waste generation that amounts, approximately, to one-third of the total global food production [5], but ensuring a production consistent with the growing demand [6]. The nutritional, environmental, and economic consequences of these dietary patterns must be taken into consideration when diet guidelines are proposed [2], and food policy, as well as production process innovation and consumers awareness, are necessary to tackle these issues. On this purpose, United Nations in the “2030 Agenda for Sustainable Development”, call for action by all countries, encouraging them to end hunger through the sustainable management of natural resources, promoting responsible consumption patterns and climate actions [7].

The demand and consumption of food within the agri-food sector are determined by many different economic, social and environmental factors. Income, population size, prices of both food and other commodities along with cultural tradition, consumers’ preferences, expectations and personal beliefs are the principal drivers of food demand responsible for shaping the consumption patterns [8]. Consumers’ preferences and choices of products and services can be explained and predicted by analysing the individual values, which constitute the background of behaviour at both conscious and non-conscious level [9]. In a context where data informative to decision makers are commonly accessible only in disparate sources, limiting their effectiveness [10], it is important to recognize the role of consumers in the evaluation of product attributes, who express their preferences considering their personal values [11]. Consumers care about the different aspects of the products they buy and many studies have identified several values that guide individual purchasing decisions: price, health, tradition, natural content, convenience and sensory appeal [9,12,13,14,15,16,17,18]. Some food values even refer to the production process and not to the product itself, such as ethical concern about environmental sustainability, animal welfare and children labour. From the literature it also emerged that food values in different countries and cultures are similar; however, they differ on the relative importance which individuals attach to them. Understanding how people choose among various products with many different characteristics is crucial in order to determine the most important features of a product. This is relevant not only for companies but also for scientists and governments when it comes to work on product innovation and packaging, to develop new technologies and advertising campaigns, or even to adopt new food policies [9].

Nowadays, the topic of the environmental burden of food production and dietary choice is becoming more widespread both within the scientific community [19,20,21,22,23] and the general public. The life cycle impact assessment results, covering multiple impacts in quantitative terms, facilitate the identification of hotspots (i.e., the main life cycle stage and activities causing significant impacts) to derive strategies for life cycle management to improve the environmental performances of product and promote the shift towards sustainable agriculture and food production systems including more sustainable food consumption patterns via environmental certification and labelling schemes [24]. Studies aimed to analyse the environmental impact of food have been conducted by many authors [23,25], but they generally focused on a single product or at least few items [5,26,27,28,29,30]. On the other hand, additional studies aimed to compare a wide variety of fresh food items, carrying out a systematic literature review of GHG emissions [2,31], but without considering the nutritional aspects. Finally, Poore & Namecek (2019), proposed a comprehensive analysis of more items, grouping them basing on their primary dietary role and benefit.

The purpose of this study is to analyse, from a life-cycle perspective, the environmental impact of meals provided in school canteens. Differently from individual foods, prepared considering the indications of nutritionists, meals served in canteens have proportionate nutritional values and are designed to guarantee a planned and balanced diet. These aspects are particularly important in school-age nutrition and represent an additional step in the analysis of the environmental impact of food.

Nowadays, the food sector is becoming increasingly global, the links among different actors of the value chain create international networks [32,33] and the production systems are more and more standardised [34]. In this context, economic, social and environmental externalities arising from the food sector spread among different countries [35]. It is highlighted that although the study was conducted at regional level in Italy, it can be seen from a general point of view since the guidelines for the composition of dishes in Italy are common at national level [36] and the considered ingredients (120) are produced in different countries. For this reason, the methodology and the results of our work could be generalizable.

## 2. Materials and Methods

Life Cycle Assessment (LCA) is an objective and standardized methodology capable of investigating the environmental behaviour of products, processes, or systems throughout their entire life cycle. As is well known, the general LCA framework is internationally defined by ISO 14040-14044 [37,38] and consists of four conceptual phases, namely, the following: (1) goal and scope definition, (2) Life Cycle Inventory (LCI), (3) Life Cycle Impact Assessment (LCIA) and (4) interpretation.

In this study, LCA methodology is applied to determine the Carbon Footprint (CF) of each canteen meal and the resulting value is then related to the food energy content. The CF (kg CO_2_ eq.), in particular, estimates the total amount of GHGs directly and indirectly emitted during the production of canteen meals, while, the food energy, expressed in kJ, is the energy released within the body when food nutrients (carbohydrates, fats and proteins) are burned [39]. The identification of these two parameters allowed us to calculate the Carbon-Footprint/Food energy (CFE) index, defined as the ratio between the CF (g CO_2_ eq./dish) and the food energy (kJ/dish). Such index provided a ranking of the most impacting meals when normalised to food energy, enabling the comparison between a large variety of foods without limiting the analysis only to their carbon intensity, but basing the evaluation of the environmental burdens in a context of balanced diet. This approach may be also employed by companies that, using the CFE index, have the possibility to communicate to the consumer an increasingly sought-after information, demonstrating their attention towards a sustainable development.

### 2.1. Goal and Scope Definition

**Figure 1:**
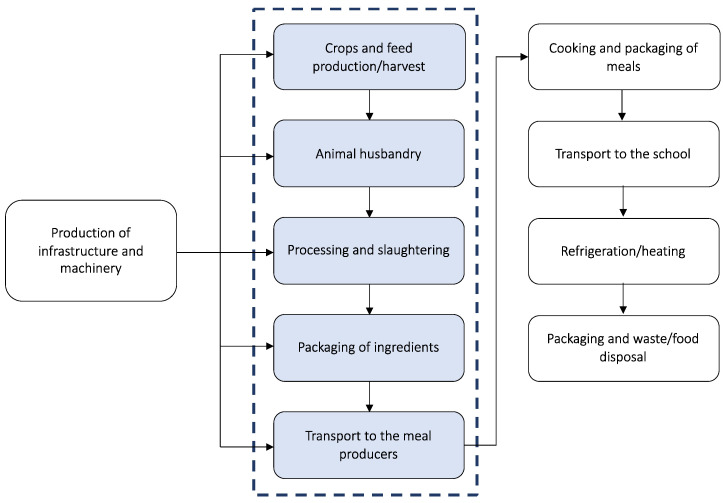
System boundaries of the study: from-cradle-to-gate approach.

The environmental impacts of meal production are evaluated with a from-cradle-to-gate approach i.e., considering from raw material production up to the transport of the ingredients to the meal producer (Figure 1). Therefore, the processes within the system boundaries are the following: (*i*) production and harvesting of crops and feed (including the use of fertilisers, energy and fuel), (*ii*) animal husbandry, (*iii*) processing and slaughtering, (*iv*) packaging and (*v*) transport to the farm that processes food and produces school meals. According to ISO 14040-14044 [37,38] guidelines, following the principle of excluding equivalent activities for LCA comparison, a cut-off criterion was applied to production of infrastructure and machinery, transport of the meals to the school, packaging end of life and waste food disposal, which are assumed to be similar for all the items. In addition, due to a lack of data and to the relatively low influence on the final value [28], system boundaries do not include the cooking of the meals and the refrigeration/heating of the meals before consumption.

The functional unit, to which the inputs and outputs of the system are related, is one dish provided to school canteens. The dishes are clustered in three sub-categories that are as follows: ‘first course’, whose principal component is normally carbohydrates; ‘second course’, mainly based on protein-rich foods, such as meat or fish; and ‘side dish’, represented by vegetables or foods high in fibres, which all together represent the complete meal for each student. The canteens have two slightly different menus, one for winter and one for summer, because, as suggested by the health care system, the seasonality of fruit and vegetables should be considered as much as possible when it comes to preparing meals for children at school [40].

No allocation criteria have been applied.

### 2.2. Inventory Phase

The list of meals and the ingredients quantities for the recipes considered in this study (Table 1) were published by the health care system of the city of Bologna (Italy) [40] according to the national guidelines, which indicates the Levels of Nutrient and Energy Reference Assumption for the Italian population [36]. Both menus contain a list of dishes for 20 days, clustered in 4 weeks (from Monday to Friday), which are repeated every month. The ingredients quantities considered in the study are those for children attending the first-grade secondary school and were taken from the same health care system of Bologna.

CFs associated to each specific ingredient are mainly collected from the international EPD system [41] and Ecoinvent 3.5 database. For nutritional value, CREA website [42] has been taken as reference. More information and data sources are reported in Table S1 of the Supplementary Materials.

Where only partial data on the agricultural stage are available, the packaging stage and the transport of the ingredients to the meal producer are added. Concerning GHG emissions, the packaging phase has been assumed to emit 0.04 g CO_2_ eq./g product, while the transport phase 0.09 g CO_2_ eq./g of product [31].

The simulation was carried out associating the specific CO_2_ eq. emissions and the energetic value of the single ingredient to their exact amount in each proposed dish. For instance, knowing the specific emissions (g CO_2_ eq./g) and the specific energetic value (kJ/g) of tomatoes (0.45; 0.80) mozzarella (8.70; 10.58) and olive oil (4.14; 37.62) and following the “Caprese salad” recipe: 100g of tomatoes, 90g of mozzarella and 6g of olive oil (Table S3 of Supplementary Materials), the total CF and energetic value referred to the single dish is computed. Then, the two obtained parameters are plotted and the CFE index is accordingly calculated.

## 3. Results and Discussion

The LCI of the study consists of 120 different foods and includes all the ingredients of the considered school menu. For each ingredient it has been calculated the CEF, which corresponds to the ratio between CF and food energy (Table S1).

In order to properly investigate the environmental impact of meals, it is necessary to first take a look at the characteristics of all the ingredients. The analysis shows that the most carbon-intensive categories are “meat and fish” and “dairy products”, whose CFs range from a minimum of 3 g CO_2_ eq./g to a maximum of 24 g CO_2_ eq./g. In particular, the highest values are attributable to veal, beef, yellowfin tuna, Parmigiano and Pecorino cheese, all of which have more than 16 g CO_2_ eq. per g of product. “Oils, spices and sauces” category has CFs between 2 and 5 g CO_2_ eq./g, while the other categories (fruit, cereals, legumes and vegetables) have a CF smaller than 2 g CO_2_ eq./g. The analysis of their CFE index (g CO_2_ eq./kJ), scatter plot shown in Figure S1 in the Supplementary Materials, indicates that almost all foods tend to align or group with the ones of the category to which they belong. In particular, “Dairy products”, “legumes” and “oils, spices and sauces” show a linear trend between CF and food energy. “Dairy products” have parameters that increase proportionally with each other, while “legumes” and “oils, spices and sauces” show a constant low CF although the energy content varies. Fruit, vegetables and cereals are grouped in areas with low environmental impact, distinguished by their food energy. “Fresh fruits” and “vegetables” have both the lowest CF and the lowest energy content (between 0 and 5 kJ/g), “cereals and pasta” are around 10–15 kJ/g, while “dry fruits” is the best category because it combines a low CF with a high food energy (25–30 kJ/g). “Meat and fish” is the only category in which it is not possible to identify a unique trend since it contains both products with low CF and high food energy (such as bacon) and products with inverse characteristics (the already mentioned veal, beef and yellowfin tuna).

**Figure 2:**
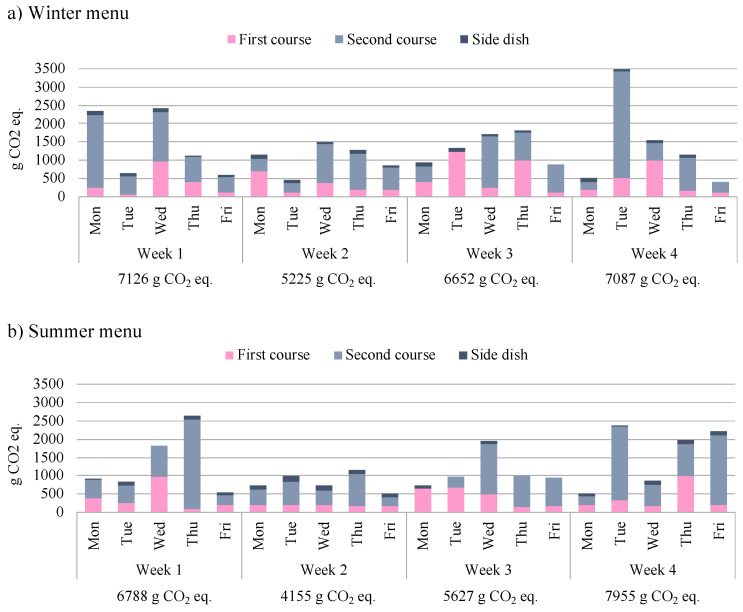
Carbon Footprint of the winter (**a**) and the summer (**b**) menu analysed.

Moving from the GHG emissions of ingredients to those of canteen meals, the results, shown in Figure 2, indicate that the CF of the winter menu is slightly higher than that of the summer menu (26.1 kg CO_2_ eq. vs. 24.5 kg CO_2_ eq.).

Analysing the differences between the two menus (full data presented in Table S2 of the Supplementary Materials), it emerges that, in winter, the consumption of meat and dairy products is higher than in summer menu, which, in turn, provides for higher amounts of vegetables, fruit, cereals and pasta. This is consistent with the guidelines of the health care system and, based on the above findings, justifies the higher CF. For the same reason, first courses are usually less impacting on the environment than second courses, as they contain less meat and dairy products but more cereal and pasta. On the other hand, being side dishes composed almost exclusively of vegetables, they resulted in the lowest environmental impact.

**Figure 3:**
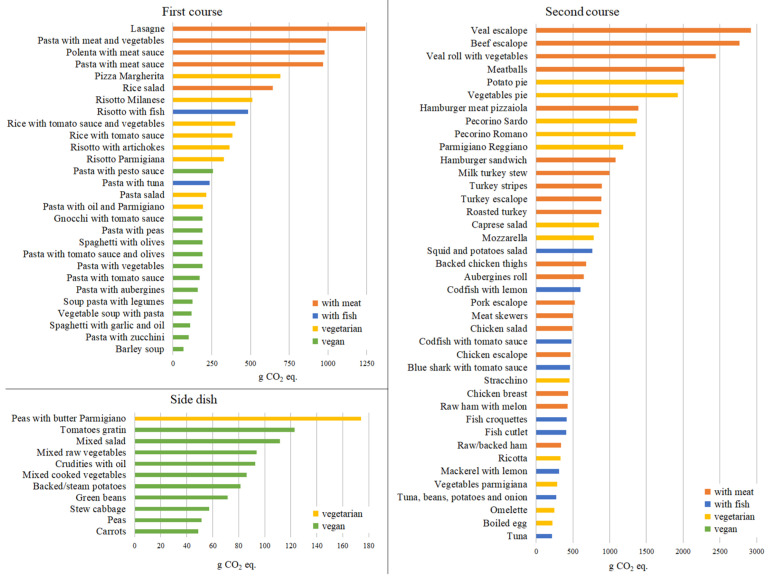
Carbon footprint (CF) of the analysed meals: first courses, second courses and side dishes.

Among the school canteen dishes, the CF of first courses ranges from 67 g CO_2_ eq. of barley soup to 1.240 g CO_2_ eq. of lasagne, the CF of second courses between tuna (not yellowfin, 215 g CO_2_ eq.) and veal escalope (2.919 g CO_2_ eq.), while side dishes GHG emissions are lower and range from 49 g CO_2_ eq. of carrots to 174 g CO_2_ eq. of peas with butter and Parmigiano cheese. The full list of all meal recipes is available in Table S3 in the Supplementary Materials. In Figure 3 all the 79 analysed dishes (28 first courses, 40 s courses and 11 side dishes) are listed by their CF and labelled according to four categories of food: with meat, with fish, vegetarian and vegan.

The results reflect the findings of the individual ingredient impacts, so dishes containing meat or fish are those that are assigned a higher CF. For instance, pasta with tomato sauce and pasta with meat sauce, which differ only by 35 g of beef meat, present a CF of 171 g CO_2_ eq. and 968 g CO_2_ eq. respectively. Within the menus, the biggest impacts (Tuesday fourth week in the winter menu and Thursday first week in the summer menu, Figure 2) come from second courses that include veal: escalope and roll with vegetables. Moreover, meat dishes tend to have a higher GHG emission than fish dishes, and dairy products (such as ripened cheeses or mozzarella) significantly increase the CF of the dishes, while vegan foods are confirmed to have the lowest impact.

**Figure 4:**
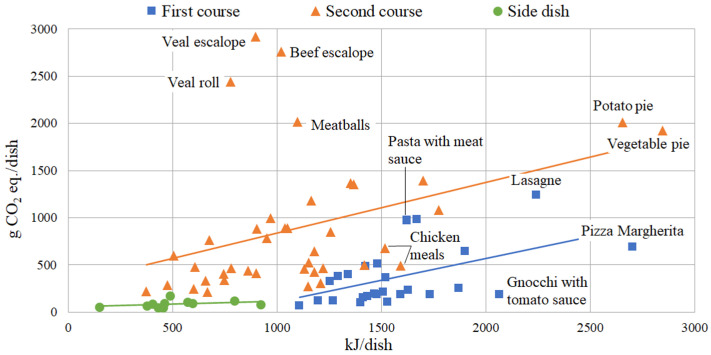
Carbon footprint (CF) of the analysed meals: first courses, second courses and side dishes.

When the CF of meals is related to their food energy, they tend to cluster as in the case of ingredients. Figure 4 allows for an easy comparison of the CF of various meals containing the same food energy, making the CFE index (g CO_2_ eq./kJ) deductible. If a meal has a low index value means that it provokes low GHG emission related to its food energy.

The graph shows that first and second courses have a direct proportionality between CF and food energy (with a few exceptions especially among second courses) with a similar trend line slope, confirming the theory described in Vieux et al., where, even if in a French context, it is found that diets with higher nutritional quality had significantly higher GHG emissions [43]. However, first courses are generally characterized by higher food energy, which, combined with the lower CF already emerged, makes the average CFE index of this category (0.23) closer to that of side dishes (0.20) than to the CFE of second courses (0.85).

Semolina pasta is the main source of food energy for first courses and the reduced use of meat ensures a low level of environmental impact. The large CF of lasagne is partially justified by the high food energy of the dish, while pasta with meat sauce (or with meat sauce and vegetables) does not compensate so well for its environmental impact. Pizza Margherita and gnocchi with tomato sauce are, among the first courses, those that best combine CF and food energy.

On the other hand, veal and beef meals (such as meatballs) are confirmed to be the worst choices since high CF is not associated with high food energy. Indeed, there are many second courses that can guarantee the same (or greater) nutrient supply to the individual and that are associated with a lower GHG emission. Concerning these, it is preferable to consume more white meat (such as chicken or turkey) than red (beef, veal, pork, lamb), both considering the environmental results of this study and for health reasons already widely documented [44]. Vegetable and potato pie values, both for food energy and CF, are consequence of the Pecorino Romano cheese with which they are made, while chicken meals are the second courses that marry the two characteristics in the best way.

The side dish category is composed for ten elevenths by vegan dishes and it shows the lowest CFE values. It is an advantage for CF, as mentioned before, but results a negative point for food energy. Among these meals there is more variability in terms of energy than impact, with baked potatoes and tomatoes gratin at the maximum and stew cabbage at the minimum.

Since part of the sourced data, for the instance the ones deriving from EPD documents, are not provided with the uncertainty, it has been chosen to not perform the uncertainty analysis.

## 4. Conclusions

In this work, GHG emissions of meals provided in school canteens have been analysed and results can be used to provide an additional supporting aspect to the costumer.

From the analysis of the ingredients, it was found that meals containing meat, fish and dairy products are the most impacting in terms of GHG emissions. Between the dairy products, cheese and butter presented the higher CF, while in the case of milk and eggs it demonstrated to be lower. In general, fresh fruit, vegetables, cereals and legumes are the foods with the lowest CO_2_ eq. contribution, although dry fruit should be considered the best option, because it combines a low CF with a high food energy (low CFE index). The first courses resulted in the best combination of the two parameters, because many of them have a high energy content that justify the environmental impact. Side dishes have an even smaller CF, but their caloric contribution to the person is also lower, resulting in a CFE index similar to that of first courses. On the contrary, second courses generally have high CF value but intermediate food energy, which makes them the most discouraged choice from an environmental/nutritional point of view.

In conclusion, results on the CF of meals and ingredients used in school canteens could be useful for further changes in school food policy in light of environmental sustainability, nutritional aspects and educational purposes and, thanks to the large ingredient inventory, the proposed one can be taken as a reference methodology for those who want to enlarge the study to the environmental impacts of different food diets or, more in general, to the food service.

A further development of this study will be aimed at extending both impact categories (such as land use and water consumption) and life cycle boundaries (such as cooking and waste disposal), providing a better knowledge about the environmental sustainability of food and meal.

## Figures and Tables

**Table 1:** List of dishes of the winter (W) and summer (S) menu of schools in the municipality of Bologna menu, reproduced from AUSL Bologna Azienda Sanitaria Locale. Indicazioni Nutrizionali per il Pasto a Scuola—Nido, Infanzia, Primaria, Secondaria; 2009 [39].

			First Course	Second Course	Side Dish
Week 1	Mon	W	Pasta with tuna	Potato pie	Mixed salad
		S	Rice with tomato sauce	Chicken salad	Carrots
	Tue	W	Barley soup	Meat skewers	Steamed potatoes
		S	Pasta with pesto sauce	Blue shark with tomato sauce	Mixed salad
	Wed	W	Pasta with meat sauce or backed pasta	Cheese	Crudities with oil
		S	Pasta with meat sauce	Caprese salad	-
	Thu	W	Rice with tomato sauce and vegetables	Backed chicken thighs	Carrots
		S	Pasta with zucchini	Loin roll with vegetables	Mixed salad
	Fri	W	Spaghetti with garlic and oil	Fish croquettes or cutlets	Peas
		S	Pasta with oil and Parmigiano	Tuna, beans, potatoes and onions	Mixed vegetables
Week 2	Mon	W	Pizza Margherita	Raw or backed ham	Mixed salad
		S	Spaghetti with olives	Raw ham with melon	Mixed salad
	Tue	W	Soup pasta with legumes	Omelettes	Backed potatoes
		S	Pasta with vegetables	Aubergines rolls	Peas with butter and Parmigiano
	Wed	W	Risotto with artichokes	Hamburger sandwich	Carrots
		S	Pasta with oil and Parmigiano	Fish croquettes	Tomatoes gratin
	Thu	W	Gnocchi with tomato sauce	Turkey stew or milk loin	Mixed raw vegetables
		S	Pasta with tomato sauce	Loin roast or cold turkey	Crudities with oil
	Fri	W	Pasta with oil and Parmigiano	Codfish with lemon	Stew cabbage
		S	Pasta with tomato sauce	Boiled eggs or omelettes	Mixed salad
Week 3	Mon	W	Rice with tomato sauce and vegetables	Chicken breast	Mixed cooked vegetables
		S	Rice salad	-	Crudities with oil
	Tue	W	Lasagne	-	Crudities with oil
		S	Pizza Margherita	Vegetables parmigiana	-
	Wed	W	Pasta with pesto	Hamburger meat pizzaiola	Peas
		S	Risotto with fish	Hamburger meat pizzaiola	Mixed cooked vegetables
	Thu	W	Pasta or polenta with meat sauce	Cheese	Carrots
		S	Pasta with aubergines	Caprese salad	-
	Fri	W	Vegetables soup with pasta	Squid and potatoes salad	-
		S	Pasta with tomato sauce	Squid and potatoes salad	-
Week 4	Mon	W	Pasta with tomato sauce and olives	Fish or tuna	Mixed raw vegetables
		S	Pasta salad	Fish or tuna	Mixed raw vegetables
	Tue	W	Risotto Milanese	Escalope	Carrots
		S	Risotto parmigiana	Meatballs	Carrots
	Wed	W	Pasta with meat sauce and vegetables	Codfish with tomato sauce	Green beans
		S	Pasta with tomato sauce	Codfish with lemon	Mixed cooked vegetables
	Thu	W	Pasta with tomato sauce	Turkey stripes	Mixed raw vegetables
		S	Pasta with meat sauce and vegetables	Turkey stripes	Mixed raw vegetables
	Fri	W	Soup pasta with legumes	Vegetables parmigiana	-
		S	Pasta with peas	Vegetable pie/omelette	Mixed raw vegetables

When the dish is not included in the menu “-” is inserted.

## Supplementary Materials

The following supporting information can be downloaded at: https://www.mdpi.com/article/10.3390/foods11020193/s1, Table S1: Carbon footprint (CF), energy content and Carbon-Footprint/Energy index (CFE index) of the food items, Table S2: Total amount of ingredients (divided by course and category) used in the menus, Table S3: Recipes from the school canteen meals, Figure S1: Carbon-Footprint/Food Energy (CFE) index graph for the analysed ingredients.
