# Supplementary material for: Environmental Impact of Meals: How Big Is the Carbon Footprint in the School Canteens?
Source: Foods. 2022 Jan 12;11(2):193. doi: 10.3390/foods11020193 (PMC8775158; doi:10.3390/foods11020193)
Supplement: Supplementary file 1 [file foods-11-00193-s001.zip › foods-1511511-supplementary.pdf]

Table S1 – Carbon footprint (CF), energy content and Carbon-Footprint/Energy index (CFE index) of the food items.

**Meat and fish**	**CF [g CO_2/g]**	**Source of CF[1]**	**Energy content[2] [kJ/g]**	**CFE index [g CO_2/kJ]**
Beef	22.80	Epd COOP, 2016	5.48	4.16
Beef (hamburger)	10.20	Epd INALCA, 2018	11.03	0.92
Blue shark	3.37	Iribarren et al., 2011	4.40	0.77
Chicken	3.65	Clune et al., 2017	7.17	0.51
Codfish	3.51	Clune et al., 2017	2.96	1.19
Cuttlefish	7.13	Clune et al., 2017	3.02	2.36
Fish (generic)	4.41	Clune et al., 2017	4.40	1.00
Mackerel	1.80	Clune et al., 2017	7.10	0.25
Mussels	9.51	Clune et al., 2017	3.51	2.71
Octopus	7.13	Clune et al., 2017	2.37	3.01
Pilchard	1.10	Clune et al., 2017	9.43	0.12
Pork (generic)	5.77	Clune et al., 2017	8.58	0.67
Pork (ham)	4.25	Bava et al., 2017	9.36	0.45
Pork (minced meat)	4.22	LCA food dk (database)	10.80	0.39
Pork (tenderloin)	4.17	LCA food dk (database)	6.58	0.63
Pork (sausages)	4.22	LCA food dk (database)	12.73	0.33
Pork (bacon)	4.23	LCA food dk (database)	22.19	0.19
Shrimps	7.80	Clune et al., 2017	2.96	2.64
Squid	7.13	Clune et al., 2017	2.84	2.51
Tuna	2.15	Clune et al., 2017	6.66	0.32
Tuna (yellowfin)	20.45	Iribarren et al., 2011	6.66	3.07
Turkey	7.17	Clune et al., 2017	5.64	1.27
Veal	24.10	Epd COOP, 2016	4.48	5.38

**Dairy, eggs, subs.**	**CF [g CO_2/g]**	**Source of CF[1]**	**Energy content[2] [kJ/g]**	**CFE index [g CO_2/kJ]**
Butter	9.25	Clune et al., 2017	31.71	0.29
Coconut milk	0.42	Clune et al., 2017	14.97	0.03
Cream	5.90	Epd Granarolo, 2018	9.74	0.61
Eggs	3.20	Epd Granarolo, 2019	5.35	0.60
Gorgonzola	10.70	Bava et al., 2017a	13.56	0.79
Mascarpone	7.11	Epd Granarolo, 2016	19.02	0.37
Milk	1.8	Epd Granarolo, 2017	2.65	0.68
Mozzarella	8.70	Epd Granarolo, 2019	10.58	0.82
Parmigiano	16.90	Bava et al., 2017a	16.61	1.02
Pecorino Romano	16.90	Vagnoni et al., 2017	17.10	0.99
Pecorino Sardo	17.10	Vagnoni et al., 2017	16.90	1.01
Rice milk	0.66	Epd OraSì, 2019	2.22	0.30
Ricotta	3.33	Epd Granarolo, 2016	6.58	0.51
Soy milk	0.69	Epd OraSì, 2019	1.33	0.52
Stracchino	5.08	Epd Granarolo, 2016	12.55	0.40
Yogurt	2.73	Epd Granarolo, 2018	2.78	0.98

**Vegetables**	**CF [g CO_2/g]**	**Source of CF[1]**	**Energy content[2] [kJ/g]**	**CFE index [g CO_2/kJ]**
Artichokes	0.48	Clune et al., 2017	1.38	0.35
Aubergines	1.35	Clune et al., 2017	0.94	1.44
Beans	0.43	Clune et al., 2017	13.62	0.03
Beans (borlotti)	1.06	Epd Valfrutta, 2019	4.26	0.25
Cowpeas	0.49	Clune et al., 2017	13.32	0.04
Broccoli	0.60	Clune et al., 2017	1.40	0.43
Cabbage	0.23	Clune et al., 2017	1.00	0.23

Carrots	0.20	Clune et al., 2017	1.71	0.12
Cauliflower	0.36	Clune et al., 2017	1.24	0.29
Celery	0.18	Clune et al., 2017	0.97	0.19
Chard	0.10	Adewale et al., 2016	1.03	0.10
Chickpeas	0.77	Clune et al., 2017	4.65	0.17
Cucumber	0.23	Clune et al., 2017	0.65	0.35
Fava beans	0.56	Ecoinvent 3.5 (database)	2.15	0.26
Fennel	0.48	Clune et al., 2017	0.62	0.77
Garlic	0.57	Clune et al., 2017	2.22	0.26
Green beans	0.31	Clune et al., 2017	1.01	0.31
Leek	0.09	de Backer et al., 2009	1.45	0.07
Legumes (generic)	0.51	Clune et al., 2017	13.56	0.04
Lentils	1.03	Clune et al., 2017	13.35	0.08
Lettuce	0.37	Clune et al., 2017	0.92	0.40
Mushrooms	0.27	Clune et al., 2017	1.28	0.21
Olives	0.63	Clune et al., 2017	10.17	0.06
Onion	0.17	Clune et al., 2017	1.18	0.14
Peas	0.38	Clune et al., 2017	3.28	0.12
Pepper	0.66	Clune et al., 2017	1.08	0.61
Potatoes	0.25	Epd Selenella, 2019	3.03	0.08
Pumpkin	0.25	Clune et al., 2017	1.23	0.20
Savoy cabbage	0.23	Clune et al., 2017	1.21	0.19
Soybean	0.49	Clune et al., 2017	18.00	0.03
Spinach	0.54	Clune et al., 2017	1.45	0.37
Sweetcorn	0.47	Clune et al., 2017	4.39	0.11
Tofu	0.49	Clune et al., 2017	3.27	0.15
Tomatoes	0.45	Clune et al., 2017	0.80	0.56
Vegetables (generic)	0.37	Clune et al., 2017	0.96	0.38
Zucchini	0.21	Clune et al., 2017	0.66	0.32

**Cereals and pasta**	**CF [g CO_2/g]**	**Source of CF[1]**	**Energy content[2] [kJ/g]**	**CFE index [g CO_2/kJ]**
Barley	0.43	Clune et al., 2017	14.47	0.03
Bread (sliced)	1.03	Epd Barilla, 2020	11.20	0.09
Breadcrumbs	1.03	Epd Barilla, 2020	14.96	0.07
Burghul	0.52	Clune et al., 2017	12.77	0.04
Corn flour	0.93	Agri-foot print (database)	14.26	0.07
Cous cous	0.52	Clune et al., 2017	15.09	0.03
Croutons	1.44	Epd Barilla, 2019	11.52	0.12
Quinoa	1.15	Clune et al., 2017	15.72	0.07
Rice	2.55	Clune et al., 2017	14.15	0.18
Seitan	0.51	Clune et al., 2017	15.49	0.03
Semolina pasta	0.82	Epd Barilla, 2019	14.26	0.06
Tagliatelle (egg pasta)	2.46	Epd De Cecco, 2017	14.49	0.17
Wheat	0.52	Clune et al., 2017	13.76	0.04
Wheat flour	0.31	Epd Molino Grassi, 2018	13.52	0.02

**Fruits**	**CF [g CO_2/g]**	**Source of CF[1]**	**Energy content[2] [kJ/g]**	**CFE index [g CO_2/kJ]**
Almonds	1.54	Clune et al., 2017	26.29	0.06
Apples	0.15	Epd Assomela, 2019	1.82	0.08
Apricot	0.43	Clune et al., 2017	1.77	0.24
Banana	0.72	Clune et al., 2017	3.19	0.23
Blueberries	0.92	Clune et al., 2017	2.07	0.44
Cashew nut	1.44	Clune et al., 2017	25.26	0.06
Cherries	0.39	Clune et al., 2017	2.01	0.19
Chestnut	0.43	Clune et al., 2017	7.28	0.06
Figs	0.43	Clune et al., 2017	2.62	0.16
Hazelnuts	0.97	Clune et al., 2017	28.08	0.03

Kiwi	0.36	Clune et al., 2017	2.02	0.18
Lemon juice	0.70	Beccali et al., 2009	0.43	1.63
Melon	0.30	Clune et al., 2017	1.43	0.21
Oranges	0.33	Clune et al., 2017	1.55	0.21
Peaches	0.43	Clune et al., 2017	1.19	0.36
Peanut	0.83	Clune et al., 2017	25.93	0.03
Pears	0.31	Clune et al., 2017	2.16	0.14
Raspberries	0.84	Clune et al., 2017	2.03	0.41
Strawberries	0.58	Clune et al., 2017	1.27	0.46
Watermelon	0.32	Clune et al., 2017	0.67	0.48
White wine	0.76	Fusi et al., 2014	2.93	0.26

**Oil, spices, sauces**	**CF [g CO_2/g]**	**Source of CF[1]**	**Energy content[2] [kJ/g]**	**CFE index [g CO_2/kJ]**
Mustard	2.09	Clune et al., 2017	24.28	0.09
Olive oil	4.14	Epd Monini, 2019	37.62	0.11
Peanuts oil	4.72	Schmidt, 2015	37.62	0.13
Pesto sauce	2.72	Epd Barilla, 2019	10.44	0.26
Ragù sauce	4.41	Epd, 2015	8.18	0.54
Saffron	3205.13	Khanali et al., 2016	13.77	232.73
Tomato sauce	1.14	Epd Valfrutta, 2019	0.89	1.28

[1] Epd source: (The International EPD® System, 2020)

[2] Data come from: Ministero delle politiche agricole, alimentari e forestali (CREA, 2019).

Table S2 – Total amount of ingredients (divided by course and category) used in the menus.

		**Winter menu**			**Summer menu**		
**Food category**	**Unit**	*First course*	*Second course*	*Side dish*	*First course*	*Second course*	*Side dish*
Meat and fish	g	170	1650	0	120	1460	0
Dairy, eggs and subs.	g	165	491	0	135	443	11
Vegetables	g	1065	560	2880	435	1465	2710
Fruits	g	0	0	0	0	300	0
Cereals and pasta	g	1465	80	0	1600	30	30
Oil, spices and sauces	g	920	200	132	974	346	90

Table S3 – Recipes from the school canteen meals.

FIRST COURSE	g	SECOND COURSE	g	SIDE DISH	g
**Barley soup**		**Aubergines roll**		**Backed/steam potatoes**	
Pearl barley	40	Aubergines	150	Potatoes	230
Potatoes	100	Ham	80	Olive oil	6
Olive oil	6	Tomato sauce	70		
		Olive oil	6	**Carrots**	
**Gnocchi with tomato sauce**				Carrots	120
Gnocchi: potatoes	250	**Backed chicken thighs**		Olive oil	6
Gnocchi: wheat flour	75	Chicken thighs	180		
Tomato sauce	70	Olive oil	6	**Crudities with oil**	
Onion	5			Fennels	100
Olive oil	6	**Beef escalope**		Carrots	100
		Beef	120	Olive oil	6
**Lasagne**		Wheat flour	10		
Egg pasta	60	Olive oil	6	**Green beans**	
Tomato sauce	70			Green beans	150
Beef meat	35	**Blue shark with tomato sauce**		Olive oil	6
Bechamel: milk	50	Blue shark	120		
Bechamel: wheat flour	50	Tomato sauce	30	**Mixed cooked vegetables**	
Olive oil	6	Olive oil	6	Spinach	30
Grated Parmigiano	5			Pumpkin	30
		**Boiled egg**		Onion	30
**Pasta salad**		Boiled egg	70	Fennel	30
Semolina pasta	80			Broccoli	30
Tomatoes	20	**Caprese salad**		Olive oil	6
Aubergines	20	Tomatoes	100		
Mozzarella	10	Mozzarella	90	**Mixed raw vegetables**	
Olive oil	6	Olive oil	6	Sweetcorn	50
				Cucumber	50
**Pasta with aubergines**		**Chicken breast**		Fennel	50
Semolina pasta	80	Chicken breast	120	Carrots	50
Aubergines	50			Olive oil	6
Olive oil	6	**Chicken escalope**			
		Chicken breast	120	**Mixed salad**	
**Pasta with oil and Parmigiano**		Wheat flour	10	Salad	50
Semolina pasta	80	Olive oil	6	Tomatoes	100
Olive oil	6			Sweetcorn	50
Grated Parmigiano	6	**Chicken salad**		Olive oil	6
		Salad	50		
**Pasta with peas**		Chicken breast	100	**Peas**	
Semolina pasta	80	Olives	30	Peas	70
Tomato sauce	70	Tomatoes	100	Olive oil	6
Peas	50	Sweetcorn	50		
Olive oil	6	Olive oil	6	**Peas with butter Parmigiano**	
				Peas	70
**Pasta with pesto sauce**		**Codfish with lemon**		Butter	5
Semolina pasta	80	Codfish	170	Grated Parmigiano	6
Pesto sauce (ready to eat)	70	Lemon juice	5		
				**Stew cabbage**	
**Pasta with ragù sauce**		**Codfish with tomato sauce**		Savoy cabbage	100

Semolina pasta	80
Tomato sauce	70
Beef meat	35
Olive oil	6

Pasta with tomato sauce

Semolina pasta	80
Tomato sauce	70
Onion	5
Olive oil	6

Pasta with tomato sauce and olives

Semolina pasta	80
Tomato sauce	70
Olives	30
Olive oil	6

Pasta with tuna

Semolina pasta	80
Tomato sauce	70
Tuna	30
Olive oil	6

Pasta with vegetables

Semolina pasta	80
Tomato sauce	70
Mixed vegetables	50
Olive oil	6

Pasta with ragù and vegetables

Semolina pasta	80
Tomato sauce	70
Beef meat	35
Mixed vegetables	50
Olive oil	6

Pasta with zucchini

Semolina pasta	80
Zucchini	50
Olive oil	6

Pizza Margherita

Wheat flour	120
Olive oil	10
Tomato sauce	80
Mozzarella	60

Polenta with ragù sauce

Polenta: corn flour	80
Polenta: olive oil	6
Tomato sauce	70
Beef meat	35

Rice salad

Rice	70
Codfish	120
Tomato sauce	30
Olive oil	6

Crud cheese (one of this)

Mozzarella	90
Ricotta	100
Stracchino	90

Fish croquettes

Codfish	80
Spinach	80
Potatoes	80
Eggs	15
Olive oil	6

Fish cutlet

Blue shark	100
Eggs	15
Olive oil	6

Hamburger meat pizzaiola

Beef hamburger	120
Tomato sauce	50
Mozzarella	10
Olive oil	6

Hamburger sandwich

Bread	60
Beef hamburger	100

Mackerel with lemon

Mackerel	170
Lemon juice	5

Meat skewers

Sausages	60
Chicken	60
Olive oil	6

Meatballs

Beef	80
Tomato sauce	40
Breadcrumbs	20
Grated Parmigiano	6
Olive oil	6

Milk turkey stew

Turkey	120
Milk	50
Butter	5

Omelette

Eggs	70
Olive oil	6
Tomato sauce	30

Tomatoes gratin

Tomatoes	150
Breadcrumbs	30
Olive oil	6

Tomatoes	30
Olives	20
Pecorino Romano	20
Backed ham	20
Peas	20
Sweetcorn	20

Rice with tomato sauce

Rice	70
Tomato sauce	50
Grated Parmigiano	6
Butter	5

Rice with tomato sauce and vegetables

Rice	70
Mixed vegetables	50
Tomato sauce	50
Grated Parmigiano	6
Butter	5

Risotto Milanese

Rice	70
Onion	5
Grated Parmigiano	6
Butter	5
Olive oil	6
Saffron	0,05

Risotto Parmigiana

Rice	70
Onion	5
Grated Parmigiano	6
Butter	5

Risotto with artichokes

Rice	70
Artichokes	30
Onion	5
Grated Parmigiano	6

**Butter**	5
Olive oil	6

Risotto with fish

Rice	70
Blue shark	30
Tomato sauce	50
Grated Parmigiano	6
Butter	5

Soup pasta with legumes

Semolina pasta	40
Beans (borlotti)	40
Potatoes	100
Olive oil	6

Pork escalope

Pork loin	120
Wheat flour	10
Olive oil	6

Potato pie

Potatoes	180
Pecorino Romano	90
Grated Parmigiano	15
Eggs	60

Raw/backed ham

Raw/backed ham	80

Raw ham with melon

Melon	300
Raw ham	80

Ripened cheese (one of this)

Pecorino Romano	80
Pecorino Sardo	80
Parmigiano Reggiano	70

Roasted turkey

Turkey	120
Olive oil	6

Squid and potatoes salad

Squid	100
Tomatoes	30
Carrots	30
Potatoes	30
Olive oil	6

Tuna

Tuna	100

Tuna, beans, potatoes and onion

Tuna	90
Beans (borlotti)	35
Potatoes	50
Onion	20
Olive oil	6

Turkey escalope

Turkey breast	120
Wheat flour	10
Olive oil	6

Turkey stripes

Turkey	120
Garlic	5
Wheat flour	10
Olive oil	6

Spaghetti with garlic and oil

Semolina pasta	80
Olive oil	10
Garlic	5

Spaghetti with olives

Semolina pasta	80
Tomato sauce	70
Olives	30
Olive oil	6

Vegetable soup with pasta

Semolina pasta	40
Mixed vegetables	100
Potatoes	100
Olive oil	6

Veal escalope

Veal	120
Wheat flour	10
Olive oil	6

Veal roll with vegetables

Veal	100
Chard	100
Olive oil	6

Vegetables parmigiana

Aubergines	60
Zucchini	60
Tomato sauce	60
Grated Parmigiano	6
Olive oil	6

Vegetables pie

Mixed vegetables	120
Eggs	60
Potatoes	180
Pecorino Romano	90
Grated Parmigiano	6
Olive oil	6

Figure S1 – Carbon-Footprint/Food Energy (CFE) index graph for the analysed ingredients.

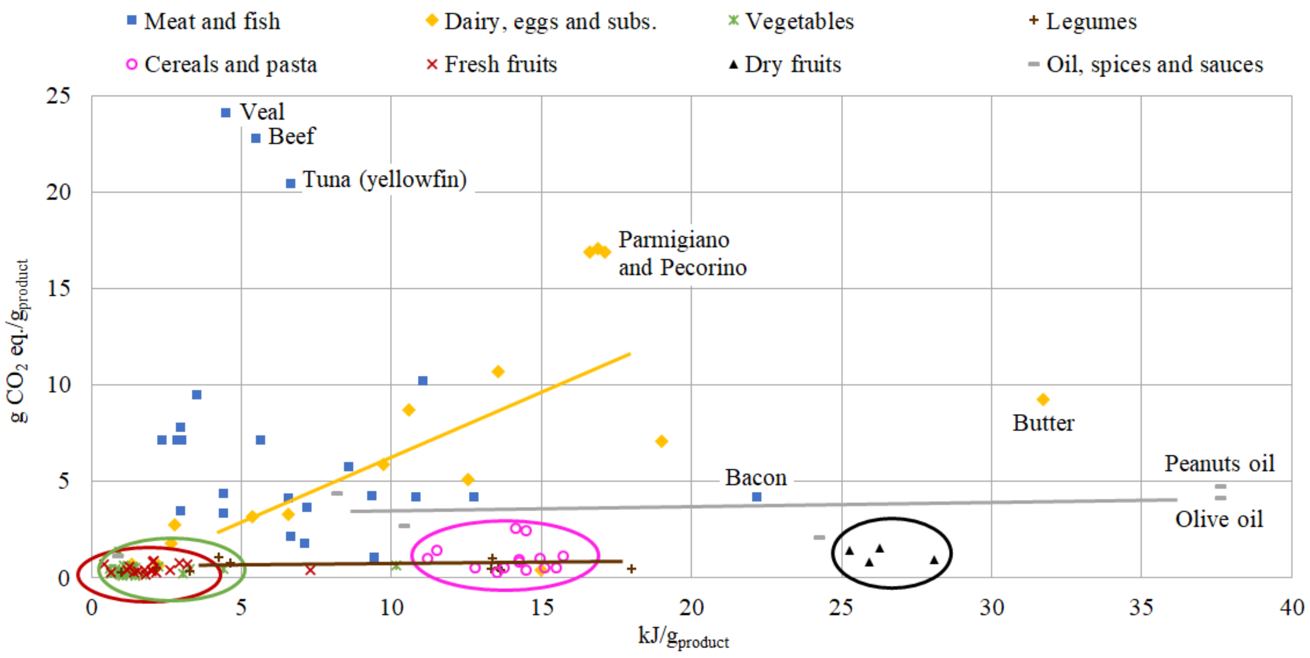

REFERENCES

Adewale, C., Higgins, S., Granatstein, D., Stöckle, C.O., Carlson, B.R., Zaher, U.E., Carpenter-Boggs, L., 2016. Identifying hotspots in the carbon footprint of a small scale organic vegetable farm. Agric. Syst. 149, 112–121. https://doi.org/10.1016/j.agsy.2016.09.004

Bava, L., Gislon, G., Bacenetti, J., Sandrucci, A., Tamburini, A., Zucali, M., 2017a. Carbon footprint of PDO cheeses: Grana Padano and Gorgonzola, in: 22nd Congress of Animal Science and Production Association. p. Ital J Anim Sci. 16, 1.

Bava, L., Zucali, M., Sandrucci, A., Tamburini, A., 2017b. Environmental impact of the typical heavy pig production in Italy. J. Clean. Prod. 140, 685–691. https://doi.org/10.1016/j.jclepro.2015.11.029

Beccali, M., Cellura, M., Iudicello, M., Mistretta, M., 2009. Resource consumption and environmental impacts of the agrofood sector: Life cycle assessment of italian citrus-based products. Environ. Manage. 43, 707–724. https://doi.org/10.1007/s00267-008-9251-y

Clune, S., Crossin, E., Verghese, K., 2017. Systematic review of greenhouse gas emissions for different fresh food categories. J. Clean. Prod. 140, 766–783. https://doi.org/10.1016/j.jclepro.2016.04.082

CREA, 2019. Consiglio per la Ricerca in agricoltura e l'analisi Economica Agraria. Tabella di composizione degli alimenti [WWW Document]. URL https://www.crea.gov.it/-/tabella-di-composizione-degli-alimenti (accessed 12.3.19).

de Backer, E., Aertsens, J., Vergucht, S., Steurbaut, W., 2009. Assessing the ecological soundness of organic and conventional agriculture by means of life cycle assessment (LCA): A case study of leek production. Br. Food J. 111, 1028–1061. https://doi.org/10.1108/00070700910992916

Fusi, A., Guidetti, R., Benedetto, G., 2014. Delving into the environmental aspect of a Sardinian white

wine: From partial to total life cycle assessment. Sci. Total Environ. 472, 989–1000. https://doi.org/10.1016/j.scitotenv.2013.11.148

Iribarren, D., Vázquez-Rowe, I., Hospido, A., Moreira, M.T., Feijoo, G., 2011. Updating the carbon footprint of the Galician fishing activity (NW Spain). Sci. Total Environ. 409, 1609–1611. https://doi.org/10.1016/j.scitotenv.2011.01.007

Khanali, M., Movahedi, M., Yousefi, M., Jahangiri, S., Khoshnevisan, B., 2016. Investigating energy balance and carbon footprint in saffron cultivation - A case study in Iran. J. Clean. Prod. 115, 162–171. https://doi.org/10.1016/j.jclepro.2015.12.013

Schmidt, J.H., 2015. Life cycle assessment of five vegetable oils. J. Clean. Prod. 87, 130–138. https://doi.org/10.1016/j.jclepro.2014.10.011

The International EPD® System, 2020. EPD Search [WWW Document]. URL https://www.environdec.com/EPD-Search/ (accessed 4.14.20).

Vagnoni, E., Franca, A., Porqueddu, C., Duce, P., 2017. Environmental profile of Sardinian sheep milk cheese supply chain: A comparison between two contrasting dairy systems. J. Clean. Prod. 165, 1078–1089. https://doi.org/10.1016/j.jclepro.2017.07.115
